# Evaluation of the Antibacterial, Anti-Cervical Cancer Capacity, and Biocompatibility of Different Graphene Oxides

**DOI:** 10.3390/molecules29020281

**Published:** 2024-01-05

**Authors:** Jorge Ivan Castro, Alana Payan-Valero, Carlos Humberto Valencia-Llano, Daniel Insuasty, Juan David Rodríguez Macias, Alejandra Ordoñez, Mayra Eliana Valencia Zapata, Jose Herminsul Mina Hernández, Carlos David Grande-Tovar

**Affiliations:** 1 Tribology, Polymers, Powder Metallurgy and Solid Waste Transformations Research Group, Universidad del Valle, Calle 13 No. 100-00, Cali 76001, Colombia; jorge.castro@correounivalle.edu.co; 2 Grupo Biomateriales Dentales, Escuela de Odontología, Universidad del Valle, Calle 4B # 36-00, Cali 76001, Colombia; alana.payan@correounivalle.edu.co (A.P.-V.); carlos.humberto.valencia@correounivalle.edu.co (C.H.V.-L.); 3 Departamento de Química y Biología, División de Ciencias Básicas, Universidad del Norte, Km 5 Vía Puerto Colombia, Barranquilla 081007, Colombia; insuastyd@uninorte.edu.co; 4 Programa de Medicina, Facultad de Ciencias de la Salud, Universidad Libre, Km 5 Vía Puerto Colombia, Barranquilla 081007, Colombia; juand.rodriguezm@unilibre.edu.co; 5 Grupo de Investigación de Fotoquímica y Fotobiología, Universidad del Atlántico, Carrera 30 Número 8-49, Puerto Colombia 081008, Colombia; maria.alejandra.ordonez@correounivalle.edu.co; 6 Grupo de Materiales Compuestos, Escuela de Ingeniería de Materiales, Facultad de Ingeniería, Universidad del Valle, Calle 13 No. 100-00, Santiago de Cali 760032, Colombia; valencia.mayra@correounivalle.edu.co (M.E.V.Z.); jose.mina@correounivalle.edu.co (J.H.M.H.)

**Keywords:** anticancer, antimicrobial, biocompatibility, Hep-2 cell line, graphene oxide, subdermal implantation

## Abstract

Cancer stands as one of the deadliest diseases in human history, marked by an inferior prognosis. While traditional therapeutic methods like surgery, chemotherapy, and radiation have demonstrated success in inhibiting tumor cell growth, their side effects often limit overall benefits and patient acceptance. In this regard, three different graphene oxides (GO) with variations in their degrees of oxidation were studied chemically and tissue-wise. The accuracy of the synthesis of the different GO was verified by robust techniques using X-ray photoelectron spectroscopy (XPS), as well as conventional techniques such as infrared spectroscopy (FTIR), RAMAN spectroscopy, and X-ray diffraction (XRD). The presence of oxygenated groups was of great importance. It affected the physicochemical properties of each of the different graphene oxides demonstrated in the presence of new vibrational modes related to the formation of new bonds promoted by the graphitization of the materials. The toxicity analysis in the Hep-2 cell line of graphene oxide formulations at 250 µg/mL on the viability and proliferation of these tumor cells showed low activity. GO formulations did not show high antibacterial activity against *Staphylococcus aureus* and *Escherichia coli* strains. However, the different graphene oxides showed biocompatibility in the subdermal implantation model for 30, 60, and 90 days in the biomodels. This allowed healing by restoring hair and tissue architecture without triggering an aggressive immune response.

## 1. Introduction

The term cancer refers to a group of more than one hundred diseases characterized by the increase of cells in a specific place in the human body, exceeding the number of normal cells, affecting the normal functioning of the tissue [1]. During the last two decades, the scientific community has developed new treatments, increasing the survival rate of affected individuals [2]. However, the methods used are characterized by being invasive, and there is a need to explore new treatment options to minimize long-term side effects and increase the outcome of therapy compared to traditional methods [3].

Cervical cancer is the second most common and aggressive gynecological malignancy and is a cause of morbidity and mortality in women worldwide [4,5]. This type of cancer is caused by persistent infection with the human papillomavirus (HPV). About 50,000 cases have been reported annually, and 31,000 have resulted in death from the disease [6]. It is believed that the occurrence and progression of this type of cancer are due to abnormal genetic and epigenetic regulation, which includes phosphorylation and acetylation of histone H3, as well as global DNA hypomethylation and hypermethylation of tumor suppressor genes [7]. To date, there are different treatments, such as radiotherapy, hormonal therapy, chemotherapy, targeted therapy, and surgical methods. The latter is the most common when the cancer is detected early [8]. Unfortunately, in most patients, its detection is done when there is significant progression; therefore, poor prognosis remains a challenge in the treatment of metastatic cervical cancer [9]. In this sense, it is necessary to unveil the mechanisms of cervical carcinogenesis as well as the exploration of new methodologies to combat the aggressiveness of cervical cancer.

Nanomaterials have been widely studied in the biomedical area due to the unique chemical, physical, and biological properties related to the dimensionality of the material. Thus, nanometric carbon exhibits low toxicity and excellent biocompatibility [10,11]. Between nanomaterials, graphene oxide (GO) has attracted much interest in contrast to some, such as silver, zinc oxide, and magnesium oxide [12]. It is a highly oxidized form of modified graphene obtained under strongly acidic conditions from graphite. GO has been explored in a wide range of medical applications, exhibiting potential in areas such as drug delivery carriers [13], biomedical imaging [14], modified tissues [15], antibacterial materials [16], and anticancer therapy [17]. Despite having demonstrated its great applicability in different biomedical fields, the systematic evaluation of the toxicological effects of graphene oxide exposure on health and the environment is still unknown. Daily human exposure to these carbonaceous materials is unavoidable; therefore, it is necessary to perform nanotoxicological studies, including safety and potential hazards.

Previous studies have shown that carbonaceous nanomaterials have complex interactions with different biological systems. However, their biocompatibility and interactions with cancer cells remain a scientific gap in which to explore new alternatives. GO has exhibited cytotoxicity against breast cancer MDA-MB-231 cells, probably due to the generation of reactive oxygen species (ROS) [18]. Additionally, GO nanolamines have shown significant toxic activity on human liver cancer HepG2 cells, related to activating the cell cycle-independent apoptotic pathway in S-phase [19]. On the other hand, different research groups have focused on the study of GO on OS cells to provide new strategies for treating this disease [20,21].

Despite the number of publications on the treatment of cancer with GO, there are certain limitations to its direct application, such as its biocompatibility and heme compatibility, colloidal stability, high selectivity towards cancer cells, and inefficient tumor uptake [22]. GO’s effect on cancer cells is complex to understand. However, the factors to consider are the material’s toxicity involving concentration, surface structure, lateral dimension, functional groups, and purity [23], which are directly related to the synthesis process. To date, there have been no studies on cytotoxic and antimicrobial using Hep-2 cancer cell lines and *S. aureus* and *E. coli*, respectively, as well as studies of biocompatibility under in vivo conditions through subdermal implantation in Wistar rats according to the degree of oxidation of the three types of graphene oxide synthesized. The results of cytotoxic, antimicrobial, and biocompatibility demonstrated the potential of the different graphene oxides synthesized represent a possible approach in the application of carbonaceous materials in the treatment against cancer. This was due to good immune response of the human body to the presence of a foreign body which caused appearance of connective tissue and decrease in immune response.

## 2. Results and Discussion

### 2.1. Characterization of Graphene Oxide

#### 2.1.1. Fourier-Transform Infrared Spectroscopy (FT-IR)

**Figure 1:**
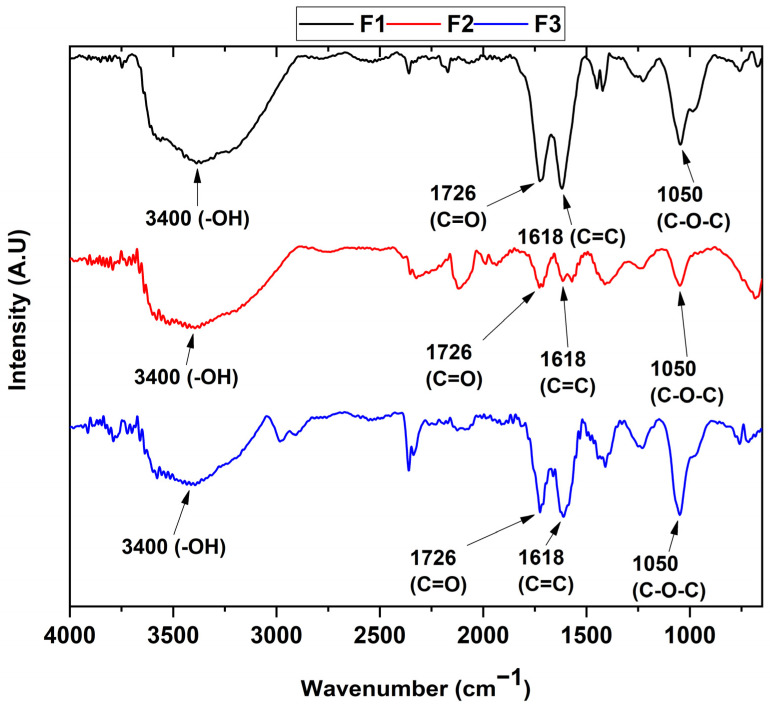
FT-IR of the different graphene oxide formulations. F1, 3 g graphite/9 g KMnO_4_/90 mL H_2_SO_4_; F2, 3 g graphite/18 g KMnO_4_/90 mL H_2_SO_4_; F3, 3 g graphite/27 g KMnO_4_/90 mL H_2_SO_4_.

The determination of the functional groups present in each of the samples can be seen in Figure 1. In the different formulations, the FT-IR spectrum reveals the presence of the vibrational modes of the -OH stretching at 3400 cm^−1^ of the COOH groups, while the bands centered at 1726 and 1618 cm^−1^ are attributed to the C=O group and the C=C bond of the aromatic rings, respectively. The band located at 1050 cm^−1^ represents the C-O-C bond stretching. Finally, the band at 2300 cm^−1^ can be attributed to atmospheric CO_2_.

**Figure 2:**
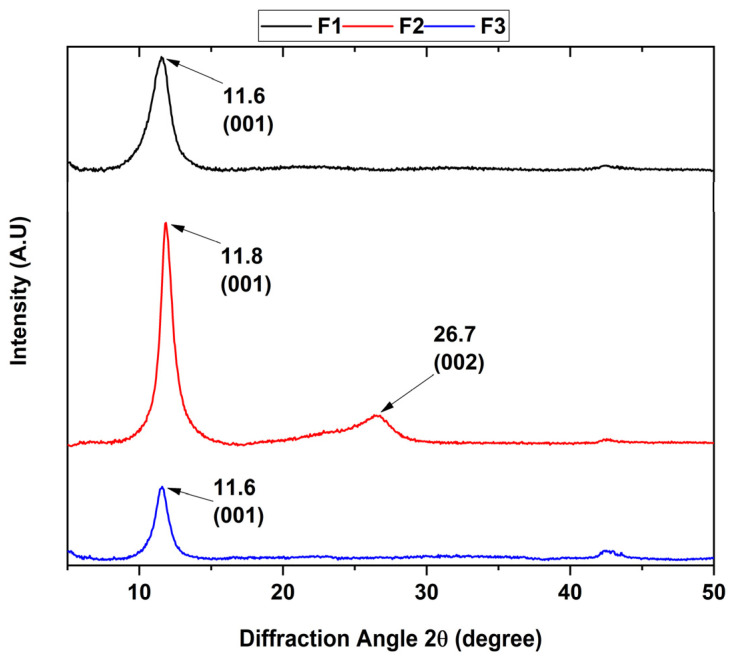
Diffractogram of the different graphene oxide formulations. F1, 3 g graphite/9 g KMnO_4_/90 mL H_2_SO_4_; F2, 3 g graphite/18 g KMnO_4_/90 mL H_2_SO_4_; F3, 3 g graphite/27 g KMnO_4_/90 mL H_2_SO_4_.

On the other hand, it can be observed that the ultrasound treatment affects the structure of the graphene oxide by reducing the oxidized groups as observed in previous studies [24], which agrees with the appearance of the diffraction peak at 26.7° for the F2 formulation in the X-ray diffraction analysis (Figure 2). Additionally, the results of the FT-IR spectra indicate that the formulations are found to have different oxidation degrees due to the change in the intensity of the C=C band at 1618 cm^−1^, as well as the change in the intensity of the C-O-C bond at 1050 cm^−1^, which are related to the formation of out-of-plane epoxide groups and lactone/ether molecules on the aromatic rings of graphene [25].

#### 2.1.2. Diffractogram for the Different Graphene Oxides

The presence of the different crystalline planes allowed the study of the crystalline phase of the various graphene oxides, where the point group was confirmed due to the position and intensity of its peaks (Figure 2). The XRD patterns clearly show that the intensity of the peak located at 26.3° characteristic of the interlamellar space of graphite attributed to the (002) plane with a hexagonal nature disappears as the degree of oxidation of the formulation increases.

Additionally, a new diffraction peak was observed at a 2θ shift of 11.6° corresponding to the (001) plane, which was attributed to the hexagonal nature of the graphene oxide. The interlaminar distance was calculated according to Bragg’s law, where a 2θ angle of 11.6 and 11.8° correspond to a distance of 7.63 and 7.48 Å, respectively. The above is because the hydrophilic functional groups located in the basal plane of GO absorb water molecules, causing the increase of the spacing d. On the other hand, it has been reported that the distance between GO layers changes according to the amount of water absorbed on the basal plane, reporting an interval of 6.1 Å for dry GO to 12 Å for hydrated GO [26].

Similarly, F2 shows a diffraction peak located at 2θ 26.7° attributed to the (200) plane characteristic to rGO with a d-spacing of 3.34 Å, which is close to the typical thickness of a pure monolayer graphene flake (3.4 Å). The XRD results indicate that the samples generally have a high degree of oxidation with a higher presence of adsorbed water for formulations F1 and F3 due to a higher increase in interlaminar distance than F2. Additionally, it is observed that F2 has reduced a portion of GO to rGO when subjected to ultrasound for 2 h.

#### 2.1.3. X-ray Photoelectron Spectroscopy

**Figure 3:**
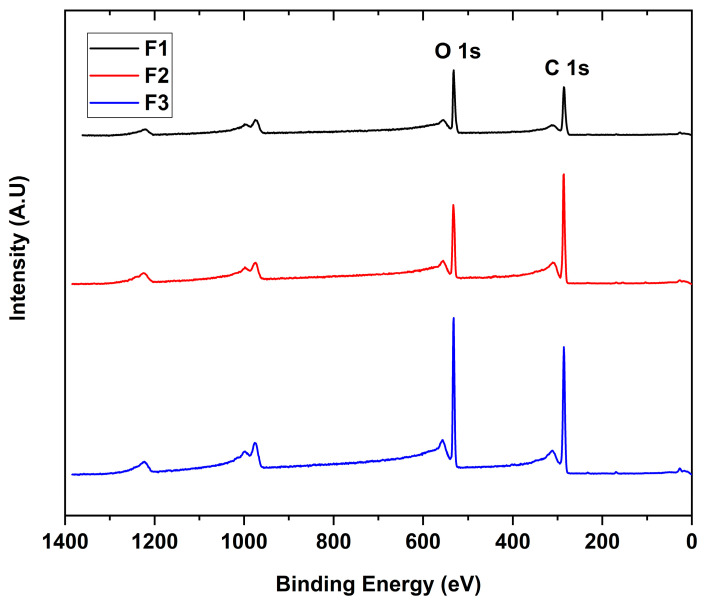
XPS survey spectra of the different GO. F1, 3 g graphite/9 g KMnO_4_/90 mL H_2_SO_4_; F2, 3 g graphite/18 g KMnO_4_/90 mL H_2_SO_4_; F3, 3 g graphite/27 g KMnO_4_/90 mL H_2_SO_4_.

The interaction between the X-rays with the different GO to give the binding energies for each functional group present in the sample is shown in Figure 3. The scanning spectra for all samples showed the presence of mainly carbon (286 eV) and oxygen (532 eV) with traces of sulfur (973 eV), probably due to contamination of the process. 

**Figure 4:**
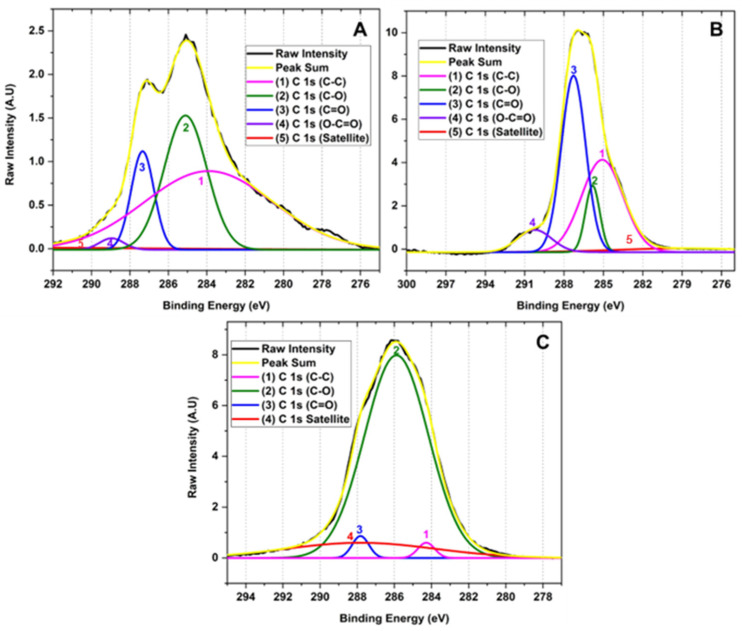
High-resolution XPS spectra for the C 1 s of for different oxidation states of GO (**A**) F1, 3 g graphite/9 g KMnO_4_/90 mL H_2_SO_4_; (**B**) F2, 3 g graphite/18 g KMnO_4_/90 mL H_2_SO_4_; (**C**) F3, 3 g graphite/27 g KMnO_4_/90 mL H_2_SO_4_.

Figure 4 shows the high-resolution XPS of the C 1 s region of the samples for the different GOs. The deconvolution of the C 1 s peak generated by the contribution of signals 1, 2, 3, 3, 4, and 5 with binding energy 283.90, 285.10, 287.34, 288.95 and 295.87 eV corresponding to C-C (3.90%), C-O (1.46%), C=O (11.79%), O-C=O (30.03%) and π-π*, satellites, respectively, for graphene oxide prepared by method F1. Similarly, the deconvolution of the C 1 s peak for the F2 method presents 1, 2, 3, and 4 signals at 278.72, 285.08, 285.82, and 287.27 eV attributed to C-C (4.96%), C-O (37.06%), C=O (9.21%), O-C=O (41.43%) and π-π* satellites. Consequently, the peak deconvolution of the C 1 s peak for the F3 method shows 1, 2, 3 signals with binding energy 284.28, 285.88, and 287.82 eV related to C-C (2.32%), C-O (81.68%), C=O (1.63%) bonds and π-π* satellites, respectively.

**Figure 5:**
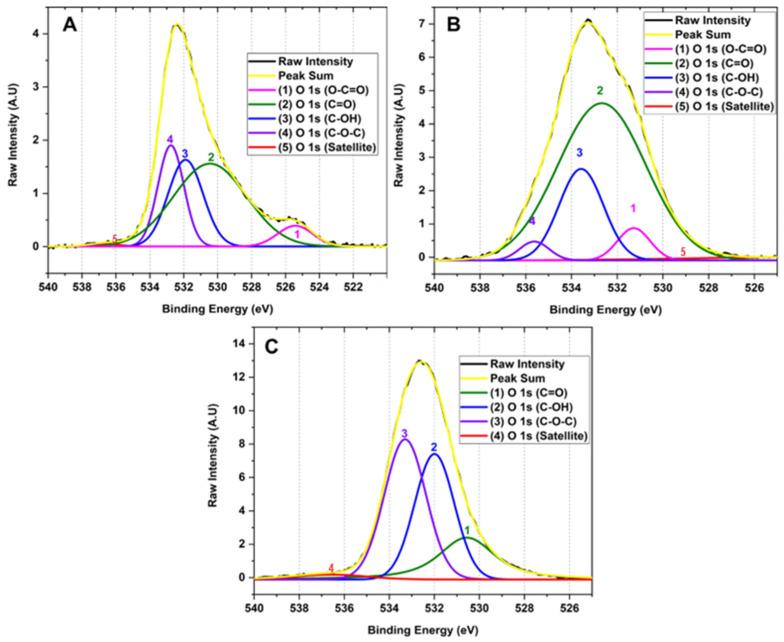
High resolution XPS spectra for the O 1 s region of the different GOs (**A**) F1, 3 g graphite/9 g KMnO_4_/90 mL H_2_SO_4_; (**B**) F2, 3 g graphite/18 g KMnO_4_/90 mL H_2_SO_4_; (**C**) F3, 3 g graphite/27 g KMnO_4_/90 mL H_2_SO_4_.

Figure 5 shows the high-resolution XPS spectrum of the O 1 s region. The deconvolution of the O 1 s region corresponds to the sum of 1, 2, 3, 3, 4 and 5 signals with binding energies at 525.42, 530.45, 531.89, 532.76 and 536.75 attributed to the functional groups O-C=O (0.67%), C=O (20.91%), C-OH (24.26%), C-O-C (48.30%) and satellites π-π*, respectively for method F1. In the same way, it is observed that method F2 has 1, 2, 3 and 4 signals that contribute to the shape of the spectrum with binding energy 523.65, 531.27, 532.67, 533.57 and 535.64 related to O-C=O (3.92%), C=O (5.18%), C-OH (68.23%), C-O-C (19.94%) and satellites π-π*. F3 presents 1, 2, 3, and 4 signals contributing to the spectrum with binding energy 530.56, 531.99, 533.30, and 536.57 corresponding to groups O-C=O (21.71%), C=O (34.82%), C-OH (41.05%) and satellites π-π*.

This type of analysis allowed us to observe that as the degree of oxidation increases, the intensity of the band related to the C-C bond decreases in comparison to the formation of new functional groups such as hydroxyl, carbonyl, and epoxy groups, clearly evidencing the formation of different degrees of oxidation in the GO. The high-resolution XPS for carbon in sample F1 showed the presence of OH groups and small bands of C=O and O-C=O groups. As the degree of oxidation increases, as observed in F2, the OH band intensity increases along with the O-C=O and C=O bands, while the band attributed to the C-C bond energy decreases. As observed in the high-resolution spectrum for oxygen in F3, a further increase in the degree of oxidation prevailed in forming C-OH, C=O, and C-O-C bonds with no ester bond formation. Additionally, it is observed that the C-O-C band prevails compared to the hydroxyl and carbonyl bands.

Therefore, XPS generally shows that hydroxyl and carbonyl groups are generated when medium concentrations of the oxidizing agent are used, as observed for formulations F1 and F2. In contrast, when the oxidizing agent concentration is increased, the formation of epoxide groups prevails, as observed for F3. This is probably because the Hummers method employs the formation of the dimanganese group (Mn_2_O_7_), resulting in a potent oxidizing agent that can epoxidize the unsaturated oxygenated groups formed during the oxidation of the graphite generating C-O-C groups as observed for F3 which contains a high level of oxidation [27,28].

#### 2.1.4. RAMAN Spectroscopy for the Different Synthesized GOs

**Figure 6:**
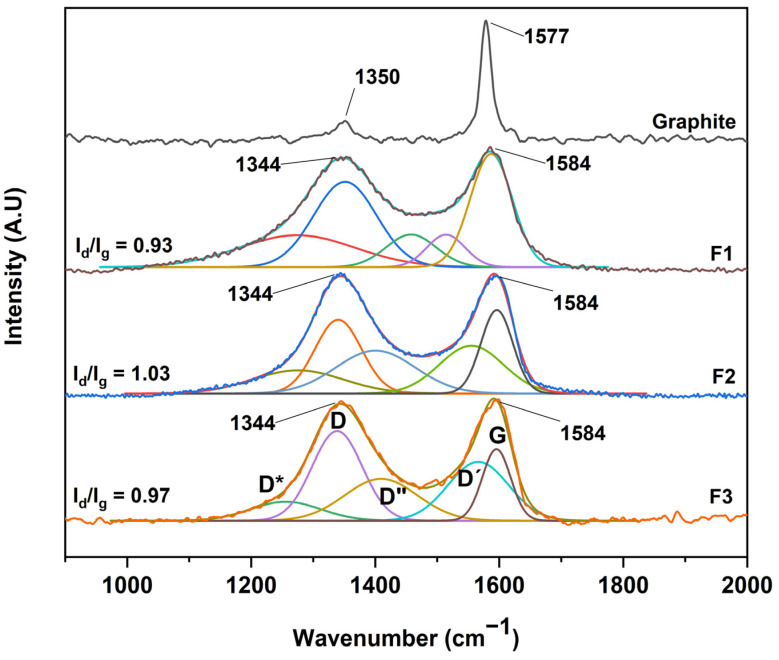
RAMAN spectra for the different graphene oxides; F1, 3 g graphite/9 g KMnO_4_/90 mL H_2_SO_4_; F2, 3 g graphite/18 g KMnO_4_/90 mL H_2_SO_4_; F3, 3 g graphite/27 g KMnO_4_/90 mL H_2_SO_4._

After the structural analysis of the oxidation degree of the different graphene oxides, a complementary study by RAMAN spectroscopy was carried out, as shown in Figure 6. No significant difference was observed between the GOs, where all spectra showed two bands, D and G, centered at 1344 cm^−1^ and 1584 cm^−1,^ respectively. However, an essential characteristic of carbon-based materials is the presence of internal bands between the D and G peaks. In this sense, the analysis by RAMAN spectroscopy and its deconvolution allows us to deepen the presence of these bands.

Different authors have fitted the D and G bands with five functions attributed to the presence of the G, D, D′, and the poorly referenced D* (1150–1300 cm^−1^) and D″ (1400–1500 cm^−1^) bands. In our case, the analysis was performed through five Gaussian functions where good coherence is shown between the mathematical parameters of linearity (R^2^) and shift square (X^2^), as shown in Table 1. Additionally, we present the sum of the five Gaussian functions, resulting in the experimental spectrum obtained by RAMAN spectroscopy.

According to the interpretation of the present bands, the D″ band is related to the amorphous phase of the crystal, where a decrease in the intensity of the material is attributed to a high crystallinity. The D* band is associated with the disordered graphitic network provided by the presence of sp^3^ hybridized carbon. Consequently, the increase in the band intensity is related to the bonding of oxygenated groups on the edges of the GO sheets. On the other hand, the D′ band is attributed to the inhomogeneity or deformation of the material. Also, the D band corresponds to the vibrational mode induced by defects or vibrations caused by imperfections due to the binding of functional groups to the basal plane of the graphitic carbon, and the G band attributed to the E_2g_ mode of first-order dispersion of graphene can be observed [26,29].

Concerning Figure 6, it can be seen that the D and G bands of the different GOs differ in three essential aspects from those of graphite: (A) the significant increase concerning the D and G band ratio (I_D_/I_G_) related to a distortion in the graphite lattice symmetry, (B) the broadening of the D and G bands attributed to the introduction of symmetry defects such as the introduction of epoxy and hydroxyl functional groups, and (C) the decrease or increase of the wavelength of the D and G band is related to the introduction of isolated double bonds in the GO.

In this sense, performing the analysis of GO purity, taking into account only the I_D_/I_G_ ratio, hinders the purity analysis as well as the graphitic level of GO. In contrast, other ratios have been proposed considering the radius of areas under the curve to estimate the degree of organization of the material. In this sense, the ratio between the areas R^2^ = D/(D + G) has been proposed, excluding bands related to poorly ordered carbons and out-of-plane defects such as tetrahedral carbons. Thus, R^2^ is more significant than 0.5 for poorly organized carbonaceous material, while R^2^ is less than 0.5 for well-organized carbonaceous material. For the GO produced in this work, the bands D*, D, D″ and D′ appear due to the synthesis process, and it is better to consider the radius of the areas R′ = (D* + D + D″ + D′)/(D* + D + D″ + D′ + G), i.e., the ratio of modes coming from defects (non-graphitic modes) to the total modes. 

According to the equation and the data presented in Table 1, ratios of 0.71, 0.81, and 0.86 were obtained for the formulations F1, F2, and F3, respectively, which suggests that the more oxidized GO is F3 due to the sp^3^ bonds and deformations produced by the oxidation which give rise to the different D bands presented in the manuscript. In addition, the G band is present, indicating a certain graphitic level, indicating that the GO is not in the form of a monolayer [29]. 

#### 2.1.5. Scanning Electron Microscopy for the Different GOs

**Figure 7:**
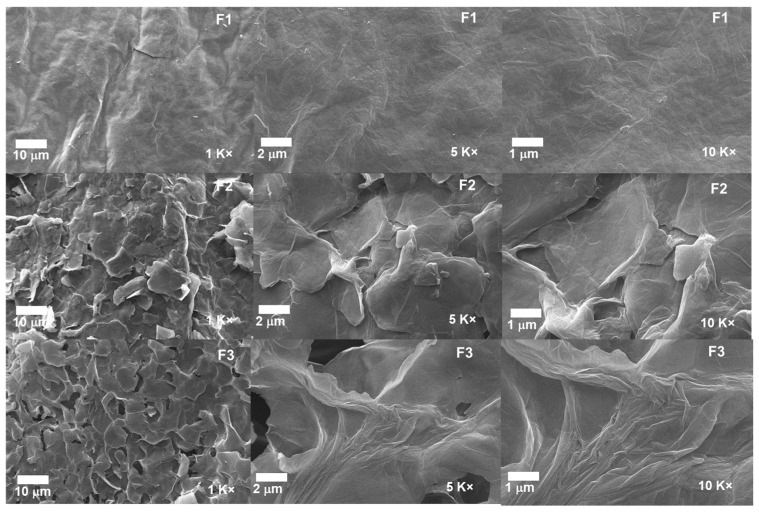
Morphology of the different GO samples. F1, 3 g graphite/9 g KMnO_4_/90 mL H_2_SO_4_; F2, 3 g graphite/18 g KMnO_4_/90 mL H_2_SO_4_; F3, 3 g graphite/27 g KMnO_4_/90 mL H_2_SO_4_.

The morphological analysis for the different GOs can be observed in Figure 7. The micrographs for the formulations F2 and F3 show a layered structure thoroughly exfoliated and separated from each other due to the different synthesis processes. In contrast, F1 shows a compact and smooth morphology, suggesting that the intercalation of oxygen groups produces the spacing by layers, the expansive effect of the temperature, and the impact of ultrasound [24].

Additionally, the introduction of KMnO_4_ at low temperatures together with H_2_SO_4_ achieves the intercalation on the graphite layers and aggregation of oxygenated groups; as the amount of KMnO_4_ increases, more oxygenated groups are added, which facilitates the intercalation of sulfuric acid into the layers and consequently the interlaminar spacing. Moreover, implementing temperature and ultrasound in the process allows the spacing to be further enlarged by disrupting the Van der Wals interaction forces between the layers [30]. The above is consistent with the XRD results, showing that the interlaminar spacing increases as the degree of oxidation of the graphite increases. 

#### 2.1.6. Test of Cytotoxic Activity of Graphene on Hep-2 Cell Line (Cervical Cancer)

**Figure 8:**
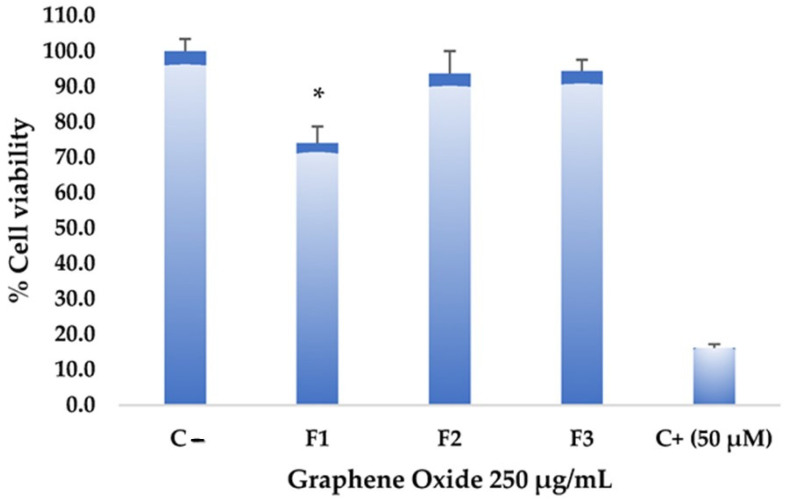
Cytotoxicity of graphene oxide formulations on the Hep-2 cell line: all assays were performed at 250 µg/mL. The positive control (C+) corresponds to Actinomycin D, while the negative control (C−) corresponds to untreated cells. * Significant difference compared to the control.

The toxicity assays on the Hep-2 cell line aim to determine the effect of graphene oxide formulations on the viability and proliferation of these tumor cells. Figure 8 shows that the different formulations did not exhibit significantly high cytotoxic effects at a high concentration of graphene oxide (250 µg/mL). However, F1 showed higher toxicity compared to the negative control. Statistically significant differences were found in F1 with a confidence level of 0.05 when analyzing variance. Despite the considerable differences from the negative control, F1 did not reach values equal to or lower than 50% cell viability, reaching only an impact percentage of 26%, so it is not considered a cytotoxic agent against this tumor cell line. Various studies have demonstrated graphene oxides’ low toxicity and high stability, making them potential candidates for drug delivery systems and promoting drug concentration in specific areas [31,32]. Substantial potential has been demonstrated in photothermal therapy, which could guide and precisely target anticancer interventions with notable specificity [33].

#### 2.1.7. Antimicrobial Activity Test of Graphene Oxides

The plate microdilution assay carried out antimicrobial activity evaluation of different graphene oxide formulations. Following incubation, absorbance was measured in each assay using a bacteria-only treatment as a reference. The absorbance values obtained in the treatments with the different formulations are generally similar to those presented in the negative control for both *S. aureus* and *E. coli*. According to the observations, these formulations cannot inhibit bacterial growth, showing very low inhibition percentages (Table 2). These findings suggest that the graphene oxide formulations tested in this study may not be effective in inhibiting the proliferation of *S. aureus* and *E. coli*. It may be necessary to conduct a more thorough analysis and explore alternative formulations or modifications to enhance the antimicrobial potential of graphene oxide. However, it is essential to note that these materials still hold significant potential for carrying specific molecules or drugs, and this aspect should not be overlooked.

#### 2.1.8. Histology (In Vivo Studies)

**Figure 9:**
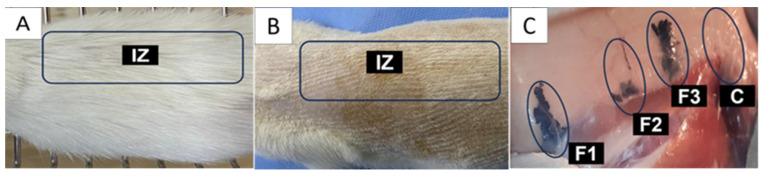
Dorsal view of a biomodel implanted for 60 days. (**A**) Skin with hair regrowth. (**B**) Skin, dorsal surface after trichotomy. (**C**) Internal surface of the skin. IZ: Implantation area. F1: Area where formulation 1 was implanted. F2: Area where formulation 2 was implanted. F3: Area where formulation 3 was implanted. C: Area where control material was implanted.

Once euthanasia was performed, a visual inspection of the implanted zones was performed. Figure 9 corresponds to the dorsal area of a biomodel after 60 days of implantation. Figure 9A shows hair recovery, while Figure 9B is the image after trichotomy, where no skin alterations attributable to the material implantation are observed. Figure 9C corresponds to the internal surface of the skin; it shows the areas where the two formulations and the control material were implanted. In none of the cases were necrotic areas or signs of infection observed.

The macroscopic image of the operated area gives a first approximation to the biocompatibility of the material; signs such as hair recovery, absence of fistulas, absence of purulent exudate, and the absence of necrotic regions in the implantation area allow us to think that the material is biocompatible.

##### Graphene Oxide Implantation Results at 30 Days

Rat skin differs histologically from human skin in the presence of the *Panniculus carnosus* muscle. In the absence of apocrine and eccrine glands, it is considered a good model for studying the healing process [34].

A wound is damage or alteration in tissue structure and function [35]; skin healing in rats is very similar to what occurs in humans but much faster; three classic phases can be identified: inflammation, proliferation, and maturation [36].

The inflammation stage coincides with the acute phase and lasts three days [36]; during this phase, immune responses and biological processes occur to heal the wound and fight any potential infection. These processes include the activation of inflammatory cells, the release of inflammatory chemicals, and the formation of blood clots to stop bleeding [35]. The proliferation phase lasts 5 to 6 days; in this period, the cells that repair the tissue begin to increase, and a new extracellular matrix is formed from granulation tissue, in which there are cells and blood vessels [35].

Finally, after six days, the maturation phase begins, which has a variable duration depending on factors such as the wound’s size, type of injury, and presence of infection, among others, in which changes occur in the scar tissue to make it stronger and more functional. After three weeks, in humans, the remodeling phase occurs in which the extracellular matrix that was formed in the healing stage is replaced by a tissue similar to the one that was lost or altered in the injury; initially, there is a connective tissue rich in collagen type III that is progressively replaced mainly through collagen type I [37].

**Figure 10:**
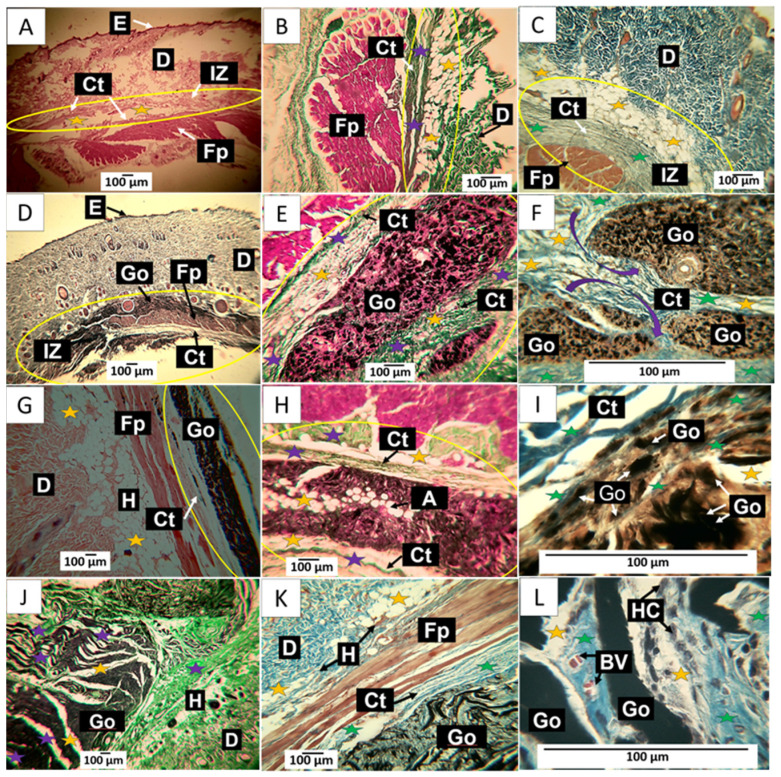
30-day implantation of the graphene oxide samples. (**A**) 4× image; HE technique. (**B**) 10× image; GT technique. (**C**) 40× image; MT technique. (**D**) 4× image; HE technique. (**E**) 10× image; GT technique. (**F**) 100× image; MT technique. (**G**) 4× image; HE technique. (**H**) 10× image; GT technique. (**I**) 10× image; HE technique. (**J**) 4× image; GT technique. (**K**) 10× image; MT technique. (**L**) 100× image; MT technique. E: Epidermis. D: Dermis. IZ: implantation zone. Ct: Connective tissue. GO: Graphene oxide. Fp: fleshy panicle muscle. Yellow oval: implantation zone. Yellow stars: adipocytes. Purple stars: type III collagen fibers. Green stars: collagen type I fibers. HC: histiocytes. BV: blood vessels. (**A**–**C**) correspond to the control formulation. (**D**–**F**) correspond to F1. (**G**–**I**) correspond to F2. (**J**–**L**) correspond to F3.

The images shown in Figure 10A–C correspond to a wound caused by the puncture of a 21-gauge syringe needle and the deposition of one cubic centimeter of physiological saline; it would be expected that after 30 days, the damage caused by the perfusion and tissue distension would have been controlled and the tissue would show normal scarring, due to the size of the lesion and the quality of the deposited material, which is biocompatible.

In Figure 10A, tissue compatible with the hypodermis is observed at a magnification of 10× between the dermis and the muscle. Using the trichromic techniques of Gomori and Masson, it is observed that the connective tissue in the scar zone is composed of type III collagen fibers (Figure 10B) and type I collagen fibers (Figure 10C). The histological observation of the implanted area indicates healing culminating in standard tissue architecture.

In Figure 10D–F corresponding to F1; it is possible to observe in the implantation zone that the material has been segmented (Figure 10D) and is surrounded by connective tissue fibers that resemble a fibrous capsule, as indicated by the purple arrows in Figure 10F. Employing the trichromic stains of Gomori and Masson, it was possible to evidence the presence of collagen fibers type III (Figure 10E) and collagen fibers type I (Figure 10F) in the capsule. Figure 10F also shows that the capsule’s collagen fibers penetrate the implantation zone, separating the material through fibrillar bundles (Figure 10F).

When reviewing what would correspond to the fibrous capsule, adipocytes are present in the fibrillar bundles that separate groups of particles. These cells can also be found in Figure 10E,F. GO particles are abundant and immersed in a connective tissue matrix surrounded by the fibrillar bundles. The presence of adipocytes in both the capsule and fibrous septum may indicate recovery of the hypodermis.

In Figure 10G–I corresponding to F2; the histological appearance differs from that reported for F1. In Figure 10G, the appearance of the GO deposit is more homogeneous, in contrast to that reported for F1; furthermore, the implantation zone is surrounded by a thin capsule composed of type III and type I collagen fibers, as seen in Figure 10H,I. Additionally, the presence of adipocytes is abundant.

The results of the implantation of the F3 formulation can be seen in Figure 10J–L. Figure 10J corresponds to a cross-section of the implantation zone. It can be seen that the connective tissue has penetrated the interior of the material, separating it into sections; in this figure, the connective tissue can be seen as white septums with some thin type III collagen fibers and abundant presence of adipocytes. Figure 10K corresponds to a section stained with Masson’s trichrome, showing type I collagen fibers surrounding the implantation zone and separating sections of the implanted material. At a magnification of 100×, it can be seen that the graphene oxide particles are immersed in a matrix of collagen with an abundance of type I collagen fibers, blood vessels, and numerous phagocytic cells. The inflammatory infiltrate is of the lymphohistiocytic type, with a predominance of histiocytes, as seen in Figure 10L.

Figure 10L shows how GO deposit phagocytosis coincides with forming a connective tissue matrix with a predominance of type III collagen.

##### Results of Material Implantation at 60 Days

**Figure 11:**
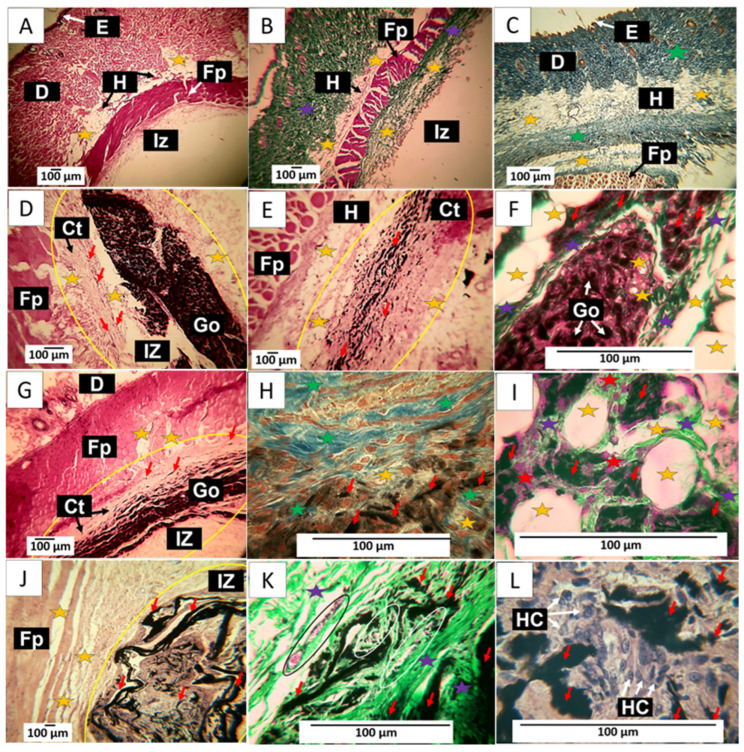
60-day implantation of the graphene oxide material. (**A**) 4× image; HE technique. (**B**) 4× image; GT technique. (**C**) 4× image; MT technique. (**D**) 10× image; HE technique. (**E**) 10× image; HE technique. (**F**) 40× image; GT technique. (**G**) 10× image; HE technique. (**H**) 40× image; MT technique. (**I**) 100× image; GT technique. (**J**) 4× image; HE technique. (**K**) 40× image; GT technique. (**L**) 100× image; HE technique. E: epidermis. D: Dermis. IZ: implantation zone. Ct: Connective tissue. GO graphene oxide. Fp: fleshy panicle muscle. Yellow oval: implantation zone. Yellow stars: adipocytes. Purple stars: type III collagen fibers. Green stars: type I collagen fibers. Red stars: Inflammatory cells. Red arrows: GO particles. White and black arrows indicate structures of interest that correspond to the related label. Black oval: Blood vessel. (**A**–**C**) correspond to control material, (**D**–**F**) to formulation 1, and (**G**–**I**) to formulation 2. (**J**–**L**) correspond to formulation 3.

The results of the implantation of the control material at 60 days show a complete recovery of the tissue. In Figure 11A, it is possible to identify the different layers of the skin, and in Figure 11B,C (processed with Gomori and Masson’s trichrome stains, respectively) indicate the presence of type III and type I collagens, which are the structural components of the extracellular matrix of skin cells.

Figure 11D–F corresponds to the implantation of F1. It is observed that at 60 days, there is more significant evidence of resorption of the material. Figure 11D shows how the material has continued to be resorbed. It highlights how the connective tissue advances as this process occurs, leaving tiny particles of the material immersed, as indicated by the red arrows. The particles in the connective tissue can be better observed in Figure 11E; some adipocytes are also visible. Figure 11F shows the presence of type III collagen fibers separating portions of the implanted material and adipose cells in its interior, unaffected by the graphene oxide.

The results of F2 can be reviewed in Figure 11G–I. Figure 11G shows a lower amount of GO compared to Figure 11D. Still, a similar process of replacement of GO by the collagen matrix is observed, in which, as the phagocytosis of GO progresses, some particles are included in the connective tissue, as can be seen in Figure 11G. Figure 11H corresponds to the same area but at a magnification of 40×. Using the MT technique, it can be observed that this connective tissue matrix is composed of type I collagen fibers and some adipocytes. Figure 11I was prepared using the GT technique. At a magnification of 100×, it can be seen that type III collagen fibers surround the adipocytes.

Figure 11J–L show the results of F3. The images correspond to a cross-section of the implantation area. Figure 11J depicts GO remnants immersed in a connective tissue matrix with brown coloration. Utilizing the GT technique, an abundance of type III collagen and several blood vessels can be observed, while in Figure 11L, at a magnification of 100×, an inflammatory infiltrate with a predominance of histiocytes can be identified as responsible for the process of phagocytosis of the GO remnants.

The histological image of these materials implanted at 60 days shows tissue in the healing process, with the implanted materials being phagocytosed and replaced by connective tissue composed of type I and III collagen. In this sense, collagen type III is replaced by connective tissue, which could indicate that, by this period, the material has been identified as compatible, and the organism can reabsorb it without the need for encapsulation. Furthermore, the presence of adipocytes in the connective tissue matrix that is being formed would indicate the recovery of the hypodermis.

##### 90-Day Material Implementation Results

**Figure 12:**
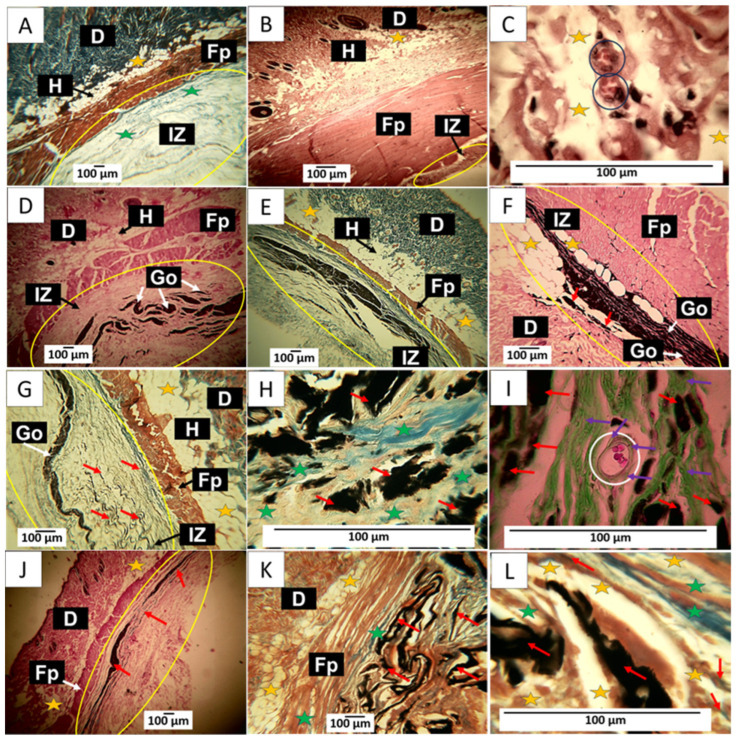
90-day implantation of the graphene oxide material. (**A**) 4× image; HE technique. (**B**) 4× image; MT technique. (**C**) 4× image; GT technique. (**D**) 4× image; HE technique. (**E**) 4× image; MT technique. (**F**) 10× image; HE technique. (**G**) 10× image; MT technique. (**H**) 100× image; MT technique. (**I**) 100× image; GT technique. (**J**) 10× image; HE technique. (**K**) 10× image; MT technique. (**L**) 100× image; MT technique. E: Epidermis. D: Dermis. IZ: implantation zone. Ct: connective tissue. GO: graphene oxide. Fp: fleshy panicle muscle. Yellow oval: implantation zone. Yellow stars: adipocytes. Green stars: type I collagen fibers. Black and white circles: blood vessels. Black and white arrows: indicate the anatomical structure related to the label. Red Arrows: GO Fragments. Purple Arrows: connective tissue. (**A**–**C**) correspond to the control material. (**D**–**F**) correspond to formulation 1, and (**G**–**I**) correspond to formulation 2. (**J**–**L**) correspond to formulation 3.

The samples implanted with the control material show for this period a very advanced recovery, as can be observed in Figure 12A–C, where the implantation zone was located after the muscle. The formation of normal connective tissue with the presence of type I collagen fibers are observed (Figure 12A). Figure 12B corresponds to a cross-section. In another biomodel, it can be observed that the implantation zone is reduced to a minimal area. Figure 12C corresponds to the exact location. Still, at a magnification of 10×, several blood vessels and numerous adipocytes in a connective tissue matrix are observed.

The implantation results of the three GO formulations show the fibrous capsule’s absence as an infiltrate. Regarding formulation F1, some remaining fragments of the material are observed immersed in connective tissue, as shown in Figure 12D. By Masson’s trichrome staining, type I collagen fibers were identified forming this connective tissue, as well as smaller fragments of the material still without resorption that were included in the connective tissue matrix (Figure 12E). Figure 12E is at a magnification of 10×. Abundant adipocytes are observed in contact with graphene oxide particles and in areas where the GO has already been resorbed.

F2 appears to have had a more rapid resorption process; there is also an absence of a fibrous capsule and inflammatory infiltrate; small amounts of remnant material are observed amid connective tissue with type I collagen fibers (Figure 12G). At 100× magnification, graphene oxide particles are observed surrounded by type I (Figure 12H) and type III collagen fibers (Figure 12I). Figure 12I also shows a blood vessel surrounded by type III collagen fibers.

Figure 12J shows that for F3, the GO remnants are lower and are immersed in the connective tissue matrix. Figure 12K corresponds to a cross-section of the implantation zone in another biomodel, showing that this connective matrix (brown in the image) also has type I collagen fibers. Figure 12L at a magnification of 100× allows us to observe numerous adipocytes occupying the matrix.

The histological images show how the graphene oxide is being phagocytosed in a time-dependent process, with significant material deposition at 30 days and a progressive decrease at 90 days. It is expected that a material implanted subdermally will induce a foreign body reaction related to how the immune system responds to foreign elements within it.

When a foreign material is introduced, the immune system identifies this material as something that is not the body’s own. This identification triggers the immune response known as the foreign body reaction, which is a defensive response aimed at protecting the body against possible damage caused by the presence of a non-biological material. Immune cells, such as macrophages and lymphocytes, may accumulate around the foreign material and attempt to eliminate or degrade it [38].

The formation of the fibrous capsule in the presence of an implanted material has been described as the last phase of a foreign body reaction; in this stage, the material is surrounded by a lax connective tissue with the presence of collagen fibers, inflammatory cells, and blood vessels, and in its periphery, it is surrounded by a network of collagen fibers. Later, once phagocytosis of the foreign material occurs, there is a phase of resolution in which the capsule disappears, and a more organized connective tissue replaces the lax tissue with a predominance of type I collagen [39].

The primary function of encapsulation is to isolate and limit the dispersion of foreign material in the surrounding tissue, thus protecting the organism. This work found histological structures compatible with a fibrous capsule only for 30 days. For formulation 1, for the other periods, the capsule has disappeared. The implantation zone was occupied by a connective tissue composed of collagen fibers type I and type III, which seems to indicate that from the second month, the intervened zones are involved in the phase of the resolution of the foreign body reaction.

## 3. Materials and Methods

### 3.1. Materials

All the materials implemented in this research are reagent-grade materials obtained from commercial sources without further purification. The graphite flake was obtained from Alfa Aesar (Tewksbury, MA, USA). KMnO_4_, H_2_SO_4_, and H_2_O_2_ were obtained from Merck (Burlington, MA, USA).

### 3.2. Synthesis of Graphene Oxide (GO)

The synthesis of GO was carried out according to previous methodologies [40]. Initially, 3 g of flake graphite was oxidized in the oxidizing mixture between KMnO_4_ and H_2_SO_4_. Then, the reaction mixture is washed with Mili Q water and centrifuged at 5000 rpm. Once washed, the GO obtained is exfoliated in an ultrasonic bath (Branson, Brookfield, CT, USA) for 2 h. Finally, the product is dried in a conventional oven for 24 h at 40 °C (Nabertherm LHT 02/18, Lilienthal, Bremen, Germany). The amounts used for each method are shown in Table 3.

### 3.3. Characterization of Graphene Oxide

#### 3.3.1. Fourier-Transform Infrared Spectroscopy (FT-IR)

The functional groups in each graphene oxide were determined through a diamond-tipped FT-IR-8400 spectrophotometer (Shimadzu, Kyoto, Japan) in a wavenumber range between 500–4000 cm^−1^.

#### 3.3.2. X-ray Diffraction (XRD)

The determination of crystallographic planes was evaluated using a PANalytical X0Pert PRO diffractometer (Malvern Panalytical, Jarman Way, Royston, UK) employing copper radiation with a wavelength of Kα1 (1.540598 Å) and Kα2 (1.544426 Å) operated in the secondary electron mode at 45 kV over a range 2θ in-between 5 and 80°.

#### 3.3.3. X-ray Photoelectron Spectroscopy (XPS)

The different energy values related to the type of bond formed on the graphene oxide surface were measured employing a Specs brand photoelectron X-ray spectrometer (Specs, Berlin, Germany) with a PHOIBOS 150 1D-DLD analyzer (PHOIBOS, Kowloon, Hong Kong, China) employing a monochromatic Al source (1486.7 eV, 13 kV, 100 W) with step energy of 20 eV. The step was 0.1 eV with 20 cycles.

#### 3.3.4. RAMAN Spectroscopy

The RAMAN-active vibrational modes attributed to the presence of sp^3^ and sp^2^ hybridized carbon were determined on a Thermo Fisher Scientific X-ray diffraction (XRD) Smart RAMAN using a 532 nm laser with a power of 6 mW (Waltham, MA, USA).

#### 3.3.5. Scanning Electron Microscopy (SEM)

Surface morphology of graphene oxides was performed through a Hitachi TM 3000 scanning electron microscope (Musashino, Tokyo, Japan) in the secondary electron mode using a 20 kV voltage accelerator with a gold layer to increase the conductivity of the samples.

Cytotoxic activity test of different graphene oxides on Hep-2 cell line (cervical cancer).

#### 3.3.6. Cultivation and Treatment Conditions

Cells were cultured at 37 °C in a humid atmosphere with 5% CO_2_. Confluence was 1 × 10^4^ cells/well, using 100 µL per replicate in a 96-well plate. Treatment was carried out with serial dilutions of 500 µg/mL, 250 µg/mL, 125 µg/mL, 62.5 µg/mL, 31.25 µg/mL, and 15.63 µg/mL of each type of graphene oxide for 24 h.

After 24 h of the treatments, the culture medium was removed and then washed with RPMI 1640 medium without fetal bovine serum to remove the graphene oxide. Once the washes were done, 90 µL of RPMI 1640 P/S 1% and 10 µL of tetrazolium bromide (MTT) at a concentration of 5 mg/mL were added at 37° C in a humid atmosphere with 5% CO_2_ and incubated for 3 h to allow the formation of formazan. At the end of the incubation time, the plates were centrifuged at 3000 RPM for 5 min. Finally, the medium was removed, and 100 µL of DMSO was added to dissolve the formazan crystals. The absorbance reading was performed in a multi-well reader (FLUOstar Omega, Ortenberg, Germany) at a wavelength of 570 nm. The results are expressed by taking as reference the control with untreated cells.

#### 3.3.7. Antimicrobial Activity Test of Graphene Oxides

Antimicrobial susceptibility tests were conducted on *E. coli* and *S. aureus* using three graphene oxide formulations at 100 µg/mL concentrations. A medium with untreated bacteria served as the negative control, while bacteria treated with amoxicillin at 100 mg/mL concentrations were used as the positive control. All tests, conducted in triplicate, were carried out in 96-well plates with a Brain Heart Infusion (BHI) culture medium. The plates were incubated for 24 h at 37 °C. After the incubation period, cell confluence was determined using a spectrophotometer at 600 nm. 

#### 3.3.8. In Vivo Biocompatibility Study of the Different Graphene Oxides

To obtain information on the material’s capacity to support and promote tissue regeneration, six five-month-old male Wistar rats, with an average weight of 380 g, were divided into three groups for observation at 30, 60, and 90 days. The analysis was carried out in triplicate, considering the implementation of the minimum number of rats and the ISO 10993-6 standard [41].

After completing implantation periods of 30, 60, and 90 days, the biomodels were euthanized through intraperitoneal injection of sodium pentobarbital/sodium diphenylhydantoin (0.3 mL per kilogram of biomodel) using Euthanex from INVET Laboratory in Cota, Colombia. Subsequently, the samples were mixed in buffered formalin for 48 h and processed following established routine procedures to generate histological sections of 6 μm [42,43]. These sections were subjected to histological staining using hematoxylin-eosin (HE), Masson’s trichrome (MT), and Gomori’s trichrome (GT) techniques. Histological images were captured using a Leica DM750 optical microscope and a Leica DFC 295 camera. The images were then processed using Leica Application Suite version 4.12.0 software (Leica Microsystem, Mannheim, Germany). All procedures adhered to the recommendations outlined in the ARRIVE (Animal Research: Reporting of In Vivo Experiments) guide. Notably, there were no biomodel deaths or post-surgical complications throughout the research. Ethical oversight was provided by the Ethics Committee with Biomedical Experimentation Animals (CEAS) in Cali, Colombia, as per Resolution No. CEAS 006-022.

#### 3.3.9. Statistical Analysis

Statistical differences for the cytotoxicity test were evaluated by an analysis of variance (ANOVA) using IBM SPSS (2022). The Tukey test was used as a post-ANOVA method to establish the differences with a significance of 5% (*p* < 0.05)

## 4. Conclusions

In this investigation, we synthesized three graphene oxides with different oxidation degrees. The presence of functional groups, such as hydroxyl and carboxyl groups, the emergence of epoxide groups, and the oxidation degree were analyzed through FTIR, XRD, RAMAN, and XPS. In the FTIR analysis, there was a noticeable increase in bands within the 1618 cm^−1^ region, corresponding to C=C bonds of the aromatic ring as well as the change in the intensity of the C-O-C bond at 1050 cm^−1^, which are related to the formation of out-of-plane epoxide groups and lactone/ether molecules on the aromatic rings of graphene. Deconvolution analysis by RAMAN spectroscopy exhibited different intensities of vibration modes related to the defects resulting from the graphitization of the material, yielding ratios of 0.71, 0.81, and 0.86 for the three types of GO, suggesting that the most oxidized material is F3. The analysis by XPS and XRD confirmed the presence of the different oxygen groups in the GO and the plane characteristic for this type of carbon material. 

Scanning electron microscopy (SEM) illustrated that a rough and exfoliated morphological structure is obtained according to the oxidation method implemented, as observed for F2 and F3. In contrast, for F1, a smooth and compacted morphology was observed. The subdermal implantation of different GOs was evaluated in a biomodel for 30, 60, and 90 days, illustrating high biocompatibility in the tissue. The toxicity assays on the Hep-2 cell line from graphene oxide formulations at 250 µg/mL on the viability and proliferation of these tumor cells demonstrated a low activity, especially for F1, which reached 26% cytotoxicity over the cell line. The GO formulations did not exhibit high antibacterial activity against *S. aureus* and *E. coli* strains.

Given the above, the remarkable healing, hair regrowth, and tissue remodeling without provoking a typical immune response gives a possible approximation of the behavior of graphene oxide-based materials, giving an idea of the possible biocompatibility of this type of material. However, the in vitro studies using Hep-2 cell lines, the antimicrobial research, and the biocompatibility analysis need complementary cytotoxicity studies with other cancer cell lines to find the possible application of these materials as an alternative to combat the disease.

## Figures and Tables

**Table 1:** Summary of the relationships between the areas of the deconvolved bands and their correlation parameters R^2^ and X^2^.

	F1		F2		F3	
Band	A	PB (cm^−1^)	A	PB (cm^−1^)	A	PB (cm^−1^)
D*	59.3	1274	33.7	1275	21.2	1256
D	86.0	1351	55.0	1340	71.8	1339
D″	26.2	1457	54.6	1400	48.8	1410
G	76.2	1587	42.3	1596	32.4	1596
D′	19.8	1514	49.1	1555	57.0	1566
A_D_/A_G_	2.646		0.852		2.267	
R^2^	0.997		0.997		0.996	
X^2^	0.00028		0.00024		0.00042	

A = area; PB = band position; R^2^ = correlation index; X^2^ = deviation.

**Table 2:** Evaluation of the antimicrobial activity of the synthesized graphene oxides using *E. coli* strains and *S. aureus*.

	Inhibition Percentages (%)		
	F1	F2	F3
*S. aureus*	8.2 ± 2.8	8.1 ± 1.7	1.0 ± 0.1
*E. coli*	4.2 ± 0.4	1.16 ± 1.0	0.6 ± 0.3

**Table 3:** Amount of reagents and times required for the GO synthesis.

Components	F1	F2	F3
Graphite (g)	3	3	3
KMnO_4_ (g)	9	18	27
H_2_SO_4_ (mL)	90	90	90
Time (h)	24	48	72

## Data Availability

Data will be made available through a request to the corresponding author. The data are not publicly available because they are required for future research. However, they can be delivered upon request to the corresponding author.
